# Testing means to scale early childhood development interventions in rural Kenya: the Msingi Bora cluster randomized controlled trial study design and protocol

**DOI:** 10.1186/s12889-019-6584-9

**Published:** 2019-03-04

**Authors:** Jill E. Luoto, Italo Lopez Garcia, Frances E. Aboud, Lia C. H. Fernald, Daisy R. Singla

**Affiliations:** 1 RAND Corporation, Santa Monica, CA USA; 2 Department of Psychology, McGill University, Montreal, Canada; 3 School of Public Health, University of California, Berkeley, USA; 4 Department of Psychiatry, Sinai Health System and University of Toronto, Toronto, Canada

**Keywords:** Early childhood development, Parenting behaviors, Village-based curriculum, Kenya, Child developmental outcomes, Community health volunteers

## Abstract

**Background:**

Forty-three percent of children under five in low and middle-income countries (LMICs) experience compromised cognitive and psychosocial development. Early childhood development (ECD) interventions that promote parent-child psychosocial stimulation and nutrition activities can help remediate early disadvantages in child development and health outcomes, but are difficult to scale. Key questions are: 1) how to maximize the reach and cost-effectiveness of ECD interventions; 2) what pathways connect interventions to parental behavioral changes and child outcomes; and 3) how to sustain impacts long-term.

**Methods:**

*Msingi Bora* (“good foundation” in Swahili) is a multi-arm cluster randomized controlled trial across 60 villages and 1200 households in rural Western Kenya that tests different, potentially cost-effective and scalable models to deliver an ECD intervention in biweekly sessions lasting 7 months. The curriculum integrates child psychosocial stimulation with hygiene and nutrition education. The multi-arm study will test the cost-effectiveness of two models of delivery: a group-based model versus a mixed model combining group sessions with personalized home visits. Households in a third study arm will serve as a control group. Each arm will have 20 villages and 400 households with a child aged 6–24 months at baseline. Primary outcomes are child cognitive and socioemotional development and home stimulation practices. In a 2 × 2 design among the 40 treatment villages, we will also test the role of including fathers in the intervention. We will estimate intention-to-treat and local average treatment effects, and examine mediating pathways using Mediation Analysis. One treatment arm will receive quarterly booster visits for 6 months following the end of the sessions. A follow-up survey 2 years after the end of the main intervention period will examine sustainability of outcomes and any spillover impacts onto younger siblings.

Study protocols have been approved by the Maseno Ethics Review Committee (MUERC) in Kenya (00539/18) and by RAND’s institutional review board. This study is funded by the National Institute for Child Health and Human Development (R01HD090045).

**Discussion:**

Results can provide policymakers with rigorous evidence of how best to design ECD interventions in low-resource rural settings.

**Trial registration:**

Clinical Trial NCT03548558 registered June 7, 2018 at clinicaltrials.gov; AEA-RCT registry AEARCTR-0002913.

## Background

### Introduction and study motivation

Vital development in language, cognitive, motor and socio-emotional domains occurs during the first few years of life, and early life outcomes are key contributors to adult outcomes such as educational achievement, labor market outcomes, and health [1, 2]. Yet more than 200 million children under age five in low and middle income countries (LMICs) will fail to reach their full developmental potential as adults, predominantly due to poverty, poor health and nutrition, and inadequate cognitive and psychosocial stimulation [3–5]. Early childhood development (ECD) interventions that integrate improved nutrition practices and child stimulation activities have been proposed as powerful policy tools for the remediation of early disadvantages in poor settings [6]. Numerous field studies have shown these programs can be effective in improving children’s developmental and health outcomes, at least in the short-term [5, 7, 8]. Moreover, early childhood is also the most cost-effective period to improve such outcomes, since this is the most critical period of brain growth and these early investments have the potential to improve adult human capital [9, 10].

Despite growing evidence on the effectiveness of early childhood stimulation interventions to improve short-term child developmental outcomes, key questions remain relating to how to make these programs scalable, and how to sustain their impacts long-term. Having a better understanding of the underlying behavioral pathways leading from intervention to parental behavior changes, and ultimately to child outcomes, is also essential to inform policy about the optimal design of interventions to maximize their scalability and sustainability.

The proposed evaluation aims to identify the most effective, cost-effective, and scalable delivery models for an ECD program designed to improve child developmental outcomes both in the short- and medium term via sustained changes in parental behaviors in rural Kenya. Our study uses a multi-arm factorial cluster randomized controlled trial (RCT) design to test the effects and cost-effectiveness of different delivery means for a curriculum designed to improve parental stimulation practices of children and the quality of the home environment that is based on previous successful ECD trials including ones conducted by our study team [11–14].

### Research questions

The questions our study addresses include the following:

#### Question 1: What is the most effective and scalable model of delivery for an integrated early childhood development intervention in a LMIC setting?

The two primary methods for delivering ECD interventions are individual home visits with mothers, or group-based meetings in a primary care or community setting. Individual home visits can offer personalized feedback, as well as individual support and problem solving to overcome personal and family barriers to behavior change, but are more expensive to implement at scale in low-income settings (Attanasio et al. [15]; Gowani, et al. [16]; Yousafzai and Aboud [17]). Group-based models in villages or clinics enjoy potential economies of scale, may modify group norms for child-rearing, and can offer mothers increased peer support, which can be a key mediator to maternal behaviors and psychological well-being in LMIC settings [12, 18]. However, groups may be comparatively weak in providing opportunities to practice and overcome personal barriers to behavior change, elements that are key for sustaining parental behavior change and improved child development outcomes long-term [7]. A mixed delivery model that combines group sessions with a limited number of personalized home visits has been hypothesized to balance the cost-effectiveness and peer support of group-based models with the benefits of personalized attention and feedback from home visits, but such a model has not yet been tested [17]. Testing the added impact and cost-effectiveness of a mixed delivery model over a group-based model will help inform how best to design ECD programs that can be implemented at scale in a LMIC setting.

#### Question 2: Does father involvement matter for the adoption of better parenting practices at home and for child development?

Successful ECD interventions have thus far focused almost exclusively on mother-child interactions. Engaging fathers in their children’s development has been suggested to be beneficial [19, 20], but the importance of fathers’ involvement in promoting ECD cognitive, language and nutritional outcomes within the household has not been formally tested in a LMIC setting. Involving fathers can potentially improve child outcomes via indirect or direct channels [21, 22]. Indirect potential benefits of father involvement include increasing the level of spouse/family-level social support or by decreasing intra-household family conflict, both of which can be protective factors in a child’s early years. Direct potential benefits of involving fathers in the intervention include the adoption of more nurturing paternal parenting practices, as well as the reduced use of harsh discipline [23–25]. Testing the added impacts from involving fathers into the intervention above and beyond the standard mother-child approach to ECD programs can inform the best strategy for maximizing effectiveness of these family-based interventions. Moreover, a planned Mediation Analysis will allow us to combine our experimental design with survey measures to examine how factors such as maternal mental health, intra-family conflict and father-child interactions mediate potential impacts.

#### Question 3: How can we achieve sustainability of impacts in a potentially scalable way?

Though there is by now a strong body of evidence that well-designed ECD programs featuring psychosocial stimulation and nutrition strategies can improve short-term child growth and developmental outcomes in LMIC settings, the evidence of medium or long-term impacts of such programs in LMIC settings is very weak. Most studies conduct only short-term evaluations of ECD interventions. To the best of our knowledge there are just 6 previous published randomized studies of psychosocial stimulation interventions in LMICs that measured effects beyond the end of the intervention period, and 5 of those 6 featured home-visiting models of delivery, which is the most expensive for scaling. The sixth study was a center-based intervention implemented in Colombia in the 1980s from which there is almost no information [26]. Out of the five home visit interventions, the best known of these is a small-scale efficacy trial in Jamaica that featured weekly home visits over 2 years among 129 stunted children beginning at ages 9–24 months [27]. Twenty years later, the intervention group had higher IQ, education levels, less violent behavior, and higher wages [2, 28, 29]. More recently, a larger study set in rural Pakistan targeted to children under age 24 months delivered by “Lady Health Workers” as part of their official government services found significant impacts from their program 12 months, 2 years and 4 years after the beginning of the interventions, although these effects tend to fade out over time [30]. At current, there is no evidence of the sustainability of a group-based ECD intervention that is also potentially scalable in very low income settings. Furthermore, there is no evidence of ECD spillover impacts onto younger siblings from a LMIC setting. Understanding spillover effects in nutrition and psychosocial stimulation interventions informs the true long-run cost-effectiveness and impacts of ECD programs [10, 31]. Finally, it is an open question what the optimal dosage is for an ECD program to ensure long-term sustained impacts while remaining cost-effective.

Our study will evaluate the sustainability of potentially scalable models of delivery in a planned, follow-up survey 2 years after the end of the main intervention period. In addition, we will test for any spillover effects on younger siblings born in the interim 2 years. Finally, between a planned endline survey and this two-year follow-up, one study arm will receive quarterly “booster” home visits 3 and 6 months following the main intervention period. These booster visits will aim to encourage sustained adherence to the behavioral changes to test the role of continued program engagement.

#### Question 4: What are the behavioral pathways through which ECD interventions may improve child developmental and health outcomes?

The evidence of short-term improvements in child cognitive, language, nutrition and health outcomes from integrated ECD interventions in LMIC settings is hampered by a lack of understanding of how exactly these interventions operate. Few studies examine the behavioral pathways leading to intervention impacts, which limits their generalizability and replicability. Though our experimental design does not directly test all potential channels through which the intervention may affect outcomes (which would be prohibitively expensive), we will combine our experiment with rich survey measures in a Mediation Analysis to begin unpacking the “black box” of how ECD interventions work [32, 33]. This approach has been increasingly used to gain greater understanding of the pathways through which ECD interventions operate to change complex behaviors [12, 13, 34, 35]. While these studies have examined how changes in behaviors mediate impacts on child outcomes, our study will expand the set of potential mediating channels to factors such as parental beliefs and expectations, mental health, and the role of intra-family dynamics and conflict. Understanding these behavioral pathways can shed light on the sustainability of any observed impacts, as well as inform the optimal design for these interventions going forward.

### Objective and hypotheses of Msingi Bora

The objective of the Msingi Bora (MB) study is to test for the most effective and cost-effective model of delivery for an integrated ECD intervention to improve child developmental outcomes among young children in rural Kenya. The design of our study will allow us to measure the relative impacts of each intervention arm compared to each other as well as to a control group. We specify the primary hypotheses of the study:

1. A delivery model that features group meetings in villages is effective in changing parental behaviors and child developmental outcomes.
2. A mixed delivery model that combines a limited number of home visits with the group meetings is more cost-effective than a model based only on group meetings to impact parental behaviours and child outcomes both at endline and follow-up 2 years later.
3. Engaging fathers in the sessions will lead to greater impacts on children’s development and sustained parental behavioral changes.
4. Adding booster home visits after the end of the 7-month intervention period will result in greater rates of sustained impacts in parental behaviors and child outcomes.

### Theory of change

In testing our study hypotheses, we will also examine our hypothesized mediating pathways through which the different interventions might affect both parental behaviors and child outcomes with the collection of intermediate indicators that comprise our theory of change. We hypothesize that Msingi Bora may operate by affecting maternal mental health, stress and conflict, self-efficacy, social support, and knowledge. Key parental behaviors potentially affected by our interventions include: i) participation in MB, ii) more effective child psychosocial stimulation practices, ii) better nutrition practices; and iii) improvements in preventive health practices such as water treatment and hand washing. We hypothesize that Msingi Bora may potentially affect child outcomes including: a) child developmental outcomes that include cognition, expressive and receptive language development, motor skills and socio-emotional development; b) child health outcomes, captured by indicators of growth and anthropometrics; and c) potential spillovers of these impacts to children that are not targeted by the intervention (younger children at home)*.*

Conceptually, all the experimental arms in our cRCT can potentially change multiple different mediators of change and through them induce changes in different types of parental behaviors and child outcomes. Of course, it is likely that we have not identified the full universe of mediators, behaviors, or outcomes affected by Msingi Bora, or that our theory of change does not correctly identify the pathways from intervention, to psychological mediator, to parental behavioral input, to child outcome. We will test our theory of change by collecting rich survey data at baseline, immediately after the intervention, as well as 2 years later in a follow-up survey.

## Methods/design

### Overview

**Fig. 1:**
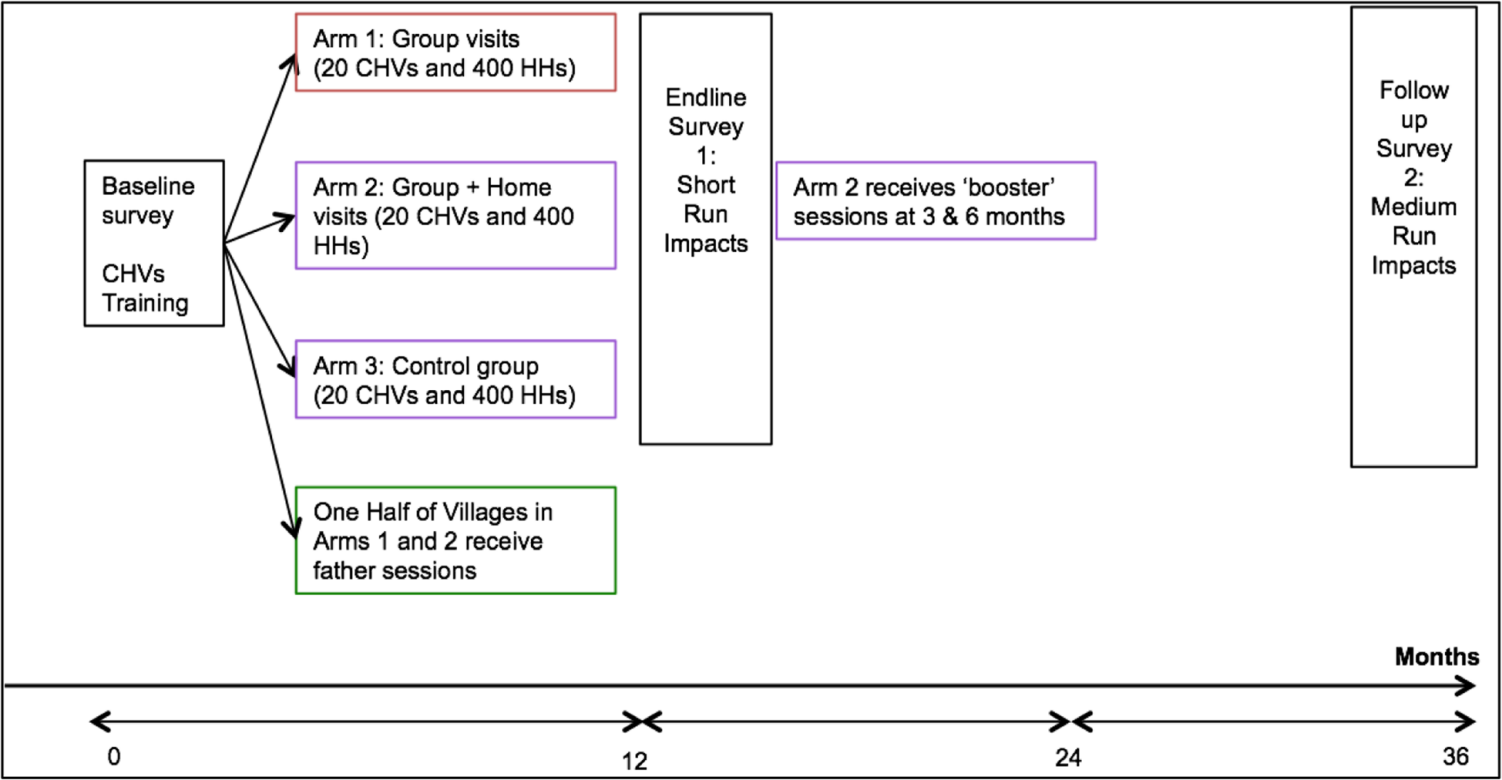
Msingi Bora Research Design and Timeline

The evaluation is a cluster randomized control trial (cRCT) across 60 villages and 1200 households with children aged 6–24 months in two counties of rural western Kenya. In collaboration with a local NGO, community health volunteers (CHVs) will be trained to implement Msingi Bora in their respective villages. The intervention will consist of 16 fortnightly sessions over 7 months to coach parents of young children on topics of nutrition, hygiene, and psychosocial stimulation strategies. Our multi-arm study will test the effectiveness and cost-effectiveness of two models of delivery for these sessions: a group-based model with mothers and their children (Arm 1–20 villages) versus a mixed model combining group sessions with personalized home visits (Arm 2–20 villages). Households in villages assigned to a third study arm will serve as a control group (Arm 3–20 villages). In a 2 × 2 design among the 40 villages assigned to a treatment arm, one half will also invite fathers to attend the 16 total sessions alongside mothers and children (20 villages total, 10 from Arm 1 and 10 from Arm 2). The other half of treatment villages will only invite mother-child dyads. Selected households for the full study will undergo baseline and endline surveys immediately before and after the 7-month intervention period to collect data on parental knowledge, beliefs, expectations about the future, social support, self-efficacy, responsive parenting practices, mother-father interactions, as well as children’s cognitive and socio-emotional developmental outcomes. Sustained changes in parental behavior and spillover impacts on younger siblings will be assessed at a second follow-up survey 2 years after the end of the intervention period. In the 2 years between the endline and follow-up surveys, households in Arm 2 (mixed model) villages will additionally receive two “booster” home visits that will come 3 and 6 months after the end of the 7-month intervention. These booster visits will reinforce Msingi Bora’s messages and aim to help families sustain any behavior changes associated with the program. Arm 1 households will not receive any further intervention after the 16 fortnightly sessions. All households, including the control group, will receive basic information about child feeding during the baseline survey. Table 1 describes the details of our research design and Fig. 1 summarizes the envisioned activities and timeline of the study.

**Table 1:** Msingi Bora Research Design and Summary of Interventions

	1. Group-only sessions	2. Group + home sessions	3. Control Arm
A. Fathers included	1A. 10 villages, 200 HHs	2A. 10 villages, 200 HHs	3. 20 villages, 400 HHs
B. Fathers not included	1B. 10 villages, 200 HHs	2B. 10 villages, 200 HHs

### Interventions

Msingi Bora’s integrated child psychosocial stimulation and nutrition and preventive health education intervention is adapted from previous successful ECD trials in LMICs conducted by members of our study team, most of which were implemented over a maximum of 6 months [11–14]. Msingi Bora’s curriculum also incorporates elements from a successful ECD intervention from Pakistan that has recently demonstrated sustained impacts [36]. The curriculum emphasizes practice, problem-solving, and peer support through the group structure, and tries to minimize didactic interactions between CHVs and parent participants.

Msingi Bora consists of 16 fortnightly sessions that will be delivered over a period of 7 months in villages assigned to a treatment arm across three subcounties (two counties) of western Kenya. The target population is families with children aged 6–24 months at baseline. Only households selected to participate in the baseline survey will be eligible to attend the village sessions in order to ensure sufficient exposure and intensity of the intervention as well as to limit the workload for the CHVs who will deliver the sessions. Mothers and their age-eligible child will be invited to participate in all sessions.

In collaboration with the local NGO Safe Water and Aids Project (SWAP), we will train 40 CHVs to implement Msingi Bora in their respective villages. The structured curriculum was piloted in April–June 2018 in six villages and the finalized curriculum includes session-specific lessons and materials with manuals for the CHVs. CHVs in villages assigned to an intervention arm will undergo 2 weeks of intensive training to deliver the curriculum, with 1 week at the beginning of the sessions and 1 week at the halfway point.

Msingi Bora’s curriculum will be delivered differently across treatment arms as follows:

#### Arm 1: Group-only sessions

For villages assigned to Arm 1, the 16 sessions will be delivered in group meetings with eligible participants within each village. Each session will last 60–90 min and has a specific curriculum designed around 5 key messages: love and respect in the family, responsive play, responsive communication, hygiene, and nutrition. The first and final sessions introduce and summarize the ECD program; four of the sessions focus on encouraging love and respect in the family, which include group discussions and role-playing activities devoted to improve maternal self-efficacy and self-esteem, practicing love and respect for children, as well as love and respect among spouses. Five of the sessions are devoted to responsive interactions between caregivers and children in play and communication, where caregivers are shown how play with children using games and materials available at home (such as a cup, bowl, and stones), as well as how to converse, sing and tell stories with the child to encourage language development. One session is devoted to encourage child health care practices including diet and hygiene, though these topics are also interspersed throughout the other sessions. Finally, every fourth session serves as a group review session at the village level (four review sessions in total). Regardless of the session topic, all sessions include 30 min devoted to mother-child interactions in play and communication activities to maximize opportunities to practice and rehearse the new behaviors in the presence of the CHV. Following this practice, participants discuss commonly expressed barriers as well as strategies to overcome barriers in their homes. Participants are encouraged to practice the new behaviors between sessions through homework.

#### Arm 2: Mixed delivery model

For villages assigned to the mixed delivery model in Arm 2, the interventions and curriculum will be identical as above for all 16 sessions with the exception that the 4 review sessions will take place during individual home visits by the CHV instead of in a group session. CHVs in these villages will be tasked with visiting each participant household during the week that a regularly scheduled fortnightly session would be held. During these home visits, CHVs will deliver identical review messages and materials, but all attention will be devoted to that mother and family and discussions about the barriers to implementing the practices, as well as feedback on behaviors and practices undertaken during these sessions, will be personalized for that household. Thus, for these review sessions, Arm 2 households receive individual home visits and Arm 1 households continue group visits; the only difference is whether on a group or individual basis.

#### Father villages

In the 20 villages where fathers are invited to participate in the intervention alongside mother-child dyads, CHVs will host separate sessions by gender for 4 of the 16 total sessions. (There will be no father-only sessions in the other 20 villages, only the mother version of these sessions.) The 4 father-only sessions will directly speak to fathers’ engagement and barriers therein in an aim to add greater social support and problem-solving abilities for whole families. These gender-separated sessions will have particular emphasis on love and respect topics in the family such as practicing respectful communication skills, but piloting showed that these topics were most easily first broached in separate meetings. The curriculum for these sessions covers interpersonal topics relevant for behavior change at the family-level, such as father involvement in child care, emotion regulation, and the types of emotional support that fathers could provide to their spouses that would be beneficial for their children’s health and development. Similar topics will be covered in the mother-only sessions for these 4, so that the curriculum and intervention is identical across mothers. Also, the remaining 12 sessions will be identical across villages, the only difference being that fathers are invited to attend these sessions alongside mothers and children.

#### Arm 2: Booster sessions

Households in Arm 2 villages will additionally receive booster home visits by the CHV 3 and 6 months following the end of the 16 fortnightly sessions. The goal of these additional booster sessions is to provide continued support to families in a cost-effective way in order to increase the potential of Msingi Bora to sustain any realized impacts in parental behavior change and child outcomes over time. These boosters will reinforce the key messages delivered during the main intervention, and will address personal barriers to sustaining the behaviors. The booster curriculum will build onto what parents have already learnt in the main program to remain age-appropriate as children age by incorporating more complex strategies to accommodate children’s growing mental capacities.

#### Arm 3: Control group

Households in villages assigned to the control group will not receive any interventions beyond the information about child feeding at the time of the baseline survey. During the study period, households in these villages will receive the status quo in terms of services from their CHV which often include reminders to attend health check-ups and vaccinations for their young children.

#### Study setting and participant eligibility criteria

This study will take place in two counties across the former Western and Nyanza Provinces, Kenya, an area characterized by high rates of poverty, child mortality, and stunting (31–34%), with the highest levels of reported spousal violence in Kenya (60%), and high levels of teenage motherhood (18%) [37]. The two counties are Homa Bay and Vihiga, within which we will work in three sub-counties of Sabatia (within Vihiga county) and East Rachuonyo and South Rachuonyo sub-counties in Homa Bay county. Sabatia sub-county has a total of 158 villages and most are predominantly Luhya-speaking. South Rachuonyo has a total of 269 villages and East Rachuonyo has 359 villages, and both are predominantly made up of Luo-speaking populations. All areas are predominantly rural, and the majority of villagers will be subsistence farmers or unskilled informal workers. The few more urban villages will not be eligible for inclusion in the study. The implementing partner SWAP has three Jamii (“community”) centers in nearby towns to the selected sub-counties that will facilitate local monitoring and supervisory capacity. All mothers or equivalent female primary caretakers aged 15 and over with a child between 6 and 24 months (classified as mature minors) will be eligible to participate in the study. If married or coupled, fathers aged 18 and over with a mother present will also be eligible to participate in surveys and the intervention as appropriate.

### Outcomes

The primary outcomes of interest of our study are child developmental outcomes and parenting behaviors including nutrition, preventive health and stimulation practices. Child developmental outcomes at endline will include cognitive, motor, language and socio-emotional development using the Bayley Scales of Infant Development 3rd edition (Bayley’s III) [38], a direct child assessment that has been validated in African settings and provides subscales for all domains of child development up to 42 months of age. At baseline we will also use the Bayley III, but only the sub-scales of cognition and language development, in order to correct for potential sample imbalances. In the follow-up survey, for those children who have aged out of the Bayley III, we will use the Malawi Developmental Assessment Tool (MDAT), which can be applied to children up to 6 years old and is culturally relevant in Sub Saharan African (SSA) settings, with good reliability [39].

Key parental stimulation behaviors will be assessed at baseline with the Family Care Indicators [40], a self-reported scale of parenting practices which measures the quality time spent with young children in learning and playing activities at home. At endline and follow-up, we will substitute the Family Care Indicators for the more comprehensive Home Observation for Measurement of the Environment (HOME) inventory, a 45 item structured survey combining mother self-report and observational items that vary by age of the child [41].

Secondary outcomes will include measures of child growth and anthropometrics, as well as parental behaviors regarding nutrition practices and preventive health behaviors. Table 2 presents a summary of the measures used for primary and secondary outcomes, measures of mediators of behavioral change, as well as measures of household socioeconomic and demographic characteristics collected in each survey wave. A household survey will be administered to the female head of household for all measures below with the following exceptions:

1. A father module will be administered to fathers or the male head of household to collect measures of paternal engagement in childcare activities, male views on intra-household decision making and conflict, as well as a measure of relationship quality from the viewpoint of the male.
2. A child questionnaire to collect the anthropometry measurements as well as direct assessment of children’s developmental outcomes using the Bayley III or MDAT, depending on the age of the child.
3. A village questionnaire will be administered to the CHVs for all sampled villages.
4. A CHV questionnaire will collect information on CHV sociodemographic characteristics, a module on their experience as a CHV, as well as measures of knowledge about ECD.

**Table 2:** Primary and Secondary Outcomes of Interest and Survey Measures (BL = baseline, EL = endline, FU = follow-up)

Outcome of interest	Measure(s)	BL	EL	FU
Primary: Child cognition, language, motor skills, socio-emotional	The Bayley Scales of Infant Development 3rd edition (Bayley’s III), which is validated in African settings and provides measures for all these dimensions of child development up to 42 months of age.	X	X	X
	The Malawi Developmental Assessment Tool (MDAT), that can be applied to children up to 6 years old, is culturally relevant in SSA settings, with good reliability (Gladstone et al. 2010) [39].			X
Primary: early childhood stimulation behaviors	The Family Care Indicators is a self-report questionnaire including questions such as how often parents take children out to the park, or other recreational activities, whether there is always an adult looking after children, the frequency of learning and play activities with children, and the amount and variety of play and learning materials (Hamadani et al. 2010) [40].	X		
	The Home Observation for Measurement of the Environment (HOME) inventory, a 45 item structured survey combining mother self-report and observational measures widely used and validated in both developed and developing countries (Bradley and Caldwell 1984) [41]. Versions of the HOME inventory have already been adapted to African settings for children up to 4 years old. For the second follow-up, we will adapt a version of the HOME-SF for parents of children of 3 to 6 years old.		X	X
Secondary: child anthropometrics	Child weight and height, and arm circumference will be measured using techniques for the WHO Multicenter Growth Reference Study (MGRS) (de Onis et al. 2004) [49].	X	X	X
Secondary: nutrition practices	Dietary diversity will be measured by maternal self-report of the foods eaten by the child in the last 24 h, following WHO recommendations about young and infant child feeding (Organization and Others 2010; Organization and UNICEF. 2003) Food security will be measured using the Household Food Insecurity Access Scale (HFIAS) (Swindale and Bilinsky 2006) [50]. A child questionnaire will be administered to all mothers including delivery information, breastfeeding history and status, and the timing of the introduction of complementary feeding.		X	X
Secondary: preventive health	A composite score of nine items including access to safe water, use of latrines, immunizations against illnesses like diphtheria, polio, tetanus, and others, deworming, etc. (Singla et al. 2015a) [13].		X	X
Mediator: Social support	We will measure perceived social support using the Lubben Social Network Scale (LSNS) which is a self-reported measure of social engagement including family and friends (Lubben et al. 2006) [51]. It consists of an equally weighted sum of 10 items used to measure size, closeness and frequency of contacts of a respondent’s social network. We will also use the Duke-UNC Functional Social Support Questionnaire (Broadhead et al. 1988) [52], which is a self-reported measure examining an individual’s perception of the amount and type of personal social support he or she receives. This scale is a multidimensional, self-administered instrument that assesses the social support that a person perceives that he or she has. The social support is measured as 2 scales for confidant or affective support. This scale has also been validated in different LMIC settings in Sub-Saharan Africa.	X	X	X
	To capture specifically the support from the spouse we will use the Relationship Support Scale that ask questions about positive and negative behaviors of husbands with wives and children. Singla et al. 2015a) [13]	X	X	X
Mediator: perceived self-efficacy	We will use the Self-Efficacy for Parenting Tasks Index-Toddler Scale or SEPTI-TS (Van Rijen et al. 2014) [53]. The SEPTI-TS enables the identification of problematic parental self-efficacy during childhood. SEPTI-TS is a 26-item questionnaire to assess parental self-efficacy in parents of toddlers. The Short Form of the SEPTI-TS showed a strong factor structure with four subscales of domain-specific parental self-efficacy (Nurturance, Discipline, Play, and Routine) that showed high reliability. Scores are rates from strongly disagree to strongly agree, and higher scores indicate stronger parental self-efficacy		X	X
Mediators: problem solving	We will adopt measures from our work in Uganda to measure ways of coping with interpersonal conflicts and daily stressors (Singla et al. 2015a) [13].	X	X	X
Mediators: mental health	Parental stress will be assessed using the Daily Stress Index (DSI) (Abidin 1990) [54]. The DSI measures on a 0–2 scale (never, sometimes, often) the difficult things that sometimes happen to people. This index has previously been used in Uganda, and the raw score will be aggregated over the 15 parts with a range of 0–30. We will measure maternal psychological well-being using the widely used Center for Epidemiologic Studies Depression Scale (CESD) scale with proven psychometric properties (Knight et al. 1997) [55].		X	X
Mediator: knowledge	Mother’s knowledge of child development will involve six questions asking mothers at what age children generally acquire social and cognitive skills (ie, recognize their mother, understand spoken words, communicate hunger, enjoy colorful moving objects, self feed, and learn things from playing with objects) (Singla et al. 2015a) [13]	X	X	X
Moderators: household socio-demographics	Socio-economic data for all households will include family composition, employment, wealth, incomes, education and housing conditions.	X	X	X
Moderators: maternal cognition	As an important predictor of child cognition, measuring maternal cognition is important to assess heterogeneous impacts by this dimension of maternal traits. While we will not measure IQ directly, we will proxy it by measuring maternal receptive language using the Peabody Picture Vocabulary Test, scale that has already been adapted to the Kenyan context (Serpell 2014) [56].		X	X
Moderators: village and CHV characteristics	Village information will include: i) access to health clinics and to primary schools, measured with distance; ii) village average socio-economic index including average employment rates; iii) prices of staple goods; iv) prices of child investment goods such as food, books, clothing, shoes, uniforms, etc.; v) data on floods and other types of weather shocks.	X	X	X

### Village and participant enrollment and randomization strategies

We will randomly assign villages and households to the various intervention components in three steps. First, we will list all potential villages in the sub-counties of Sabatia (Vihiga county), and East and South Rachuonyo (Homa Bay county) that satisfy three requirements: 1) There are estimated to be at least 20 households with children that will be between 6 and 24 months old at the time of the baseline survey based on village lists collected semiannually by CHVs as part of their regular duties; 2) There is at least one CHV assigned to that village who can be trained into the study curriculum; and 3) Villages will be sufficiently geographically dispersed (> 1.5 km distance) so that households within villages assigned to the control arm do not travel to access the intervention in treatment villages. In some cases, we may merge 2 or 3 neighboring villages with the assistance of local Community Health Units in order to reach the estimated size of 20 children in our age range. From the pool of eligible villages, merged and non-merged, we will randomly select 60 villages to participate in the full study stratified by sub-county (20 villages per sub-county). The villages will form our study’s clusters for subsequent randomization to intervention arm.

Second, within each selected village, a field interviewer will work with the village CHVs to conduct a census to identify all households with a child aged 6–24 months as of baseline. During this census, when an age-eligible child is identified, interviewers will secure informed consent for participation by the primary caretaker for that child and her spouse (if present), as well as secure GPS coordinates for the household. If this list of consenting households is greater than the 20 needed for our study’s sample size calculations, we will randomly draw a sample of 20 households using a random number generator and this process will be made clear to households during informed consent procedures. A separate team of trained interviewers will then conduct a baseline survey on the final sample of 20 consenting households within each of the 60 villages, using the collected GPS coordinates to locate the households and collecting the measures outlined in Table 2.

Third, after the baseline surveys are complete, we will randomly assign CHVs and their villages to one of three study arms using a random number generator. Each arm will have 20 CHVs and 400 households. After assignment to study arm, villages assigned to Arms 1 or 2 will undergo a secondary randomization procedure to determine those villages that additionally will invite fathers to the sessions (in a 1:1 ratio among the 40 villages assigned to Arms 1 and 2). All randomizations will be stratified by Sub-county to ensure balance across treatment arms on any village-level characteristics that have the potential to have some relationship with intervention effects.

CHVs in villages assigned to an intervention arm will attend the training course described above. We will pay all CHVs a stipend for their collaboration in the census and the intervention as appropriate.

#### Blinding

Our study will have separate teams for collection of surveys and program implementation. Interventions will be coordinated by the local NGO SWAP through a team including a Study Supervisor and 6 project monitors that will supervise and monitor the work of the CHVs. Survey data collection will be conducted by an external team of qualified enumerators and supervisors from the Kenya Medical Research Institute (Kemri). Due to the nature of the intervention, the participants and delivery agents will not be blinded to their study allocation as part of the program implementation team. Data collectors of surveys for the research team will, however, be blinded to the intervention allocation status of participants and villages. Baseline surveys will be collected prior to randomization. Likewise, data analysis will be blinded to the intervention status of participants and villages.

#### Compliance

We do not anticipate noncompliance with treatment status for those villages and households assigned to a control arm because our sampling frame will ensure a minimum distance between villages and CHVs included in the study. For individual households in villages assigned to a treatment arm, our power calculations presented below account for noncompliance with treatment by including an expected attendance rate of 75% to the sessions.

#### Retention

Once a mother-child dyad is enrolled into the study, we will make every reasonable effort to follow the dyad (as well as father, if applicable) for the entire study period. The baseline survey will collect mobile phone numbers for household members to facilitate follow-up surveys as well as invitations to attend sessions, if appropriate. The mobile number of one neighbor will additionally be collected to help identify cases of non-retention. Reasons for non-retention (e.g., loss to follow-up) will be recorded. At each survey round we will make up to 3 attempts to contact a household for resurveying prior to dropping from the sample. Our power calculations account for up to 15% attrition to allow for such instances.

### Data

#### Sample size and power calculations

The sample of 60 villages and 20 households per village for the full evaluation is calculated for measures of child developmental outcomes that will be measured using the Bayley III scale taken at the endline survey. This scale has a usual mean of 100 with a standard deviation (SD) of 15 [42]. Our previous work in Uganda has an effect size of 0.36 SD [18]. Our previous work in this part of Kenya has found annual attrition rates of roughly 15% and we found roughly 80% compliance in a previous collaboration in the study area on a similar population of mothers with young children [43]. We conservatively assume 75% compliance among those invited to the sessions, 15% attrition, and an ICC of 0.07 within 60 CHV catchment areas. In a side-by-side comparison between study arms our sample size of 400 mother-child dyads in each arm provides 80% power to detect an increase in children’s cognitive and receptive language development of 0.30 SD at the 5% level of statistical significance. The impact from involving fathers has similar power. In a 2:1 test comparing the two treatment arms and the control arm we can detect a 0.26 SD effect size under similar assumptions. To detect spillovers effects in younger siblings at the second follow-up impacts survey we estimate that roughly 75% of households will have a younger sibling (average parity is about 5.4 children per mother in Nyanza [37]), implying we can detect 0.33 SD effects in comparing siblings of treated vs. untreated children at the second follow-up survey.

#### Data sources and procedures

Survey data will be collected with SurveyCTO on Android tablets for the measures enumerated above. Baseline data collection is estimated to take roughly 2 months. Data collection will terminate upon reaching our target sample size at baseline. All households surveyed at baseline will be re-contacted to undergo an endline survey roughly 10–12 months later. Duration, procedures and measures will be identical to baseline. We will make up to 3 attempts to contact a household for resurveying prior to dropping from the sample. The interviewer will reassess the child assessed at baseline and interview the mother (and the father as appropriate). Roughly 2 years after the endline survey, all households will again be re-contacted to undergo a follow-up survey. Measures and procedures will be similar to the endline survey. However, for those mothers that have a new child aged 6–24 months at this time point, we will additionally assess these younger siblings using the same measures and procedures as used on their older siblings at earlier waves.

##### Monitoring and process data

Monitoring data in the form of attendance sheets and monitoring checklists will be collected by SWAP supervisory staff from the 3 Jamii centers during each village session over the 8-month intervention period. The data will be collected using SurveyCTO and will be transmitted to SWAP servers in Kisumu, where SWAP staff will clean and aggregate the data to be transferred to an Aggregate Server hosted at RAND.

##### Costing data

The total costs for the interventions include the wages of the CHV, the training costs per CHV, and the wages, training, and supervision costs for SWAP management as well as the information and communication materials that comprise the intervention. During implementation of the study, SWAP supervisory staff will be asked to record actual expenditures incurred. In addition, we will collect information about private costs to mothers and fathers for attendance such as transportation costs as well as estimates about the opportunity costs of attendance.

##### Data management plan

Survey data will be collected on tablets using SurveyCTO and will be treated with the maximum norms of confidentiality following the study protocols involving human subjects reviewed by the RAND Human Subjects Protection Committee (HSPC) as well as the Maseno University Ethical Review Committee (MUERC). Data will be uploaded to an Aggregate Server hosted at RAND with password protected files. Participant names will be removed from the data and no longer stored in any table after the successful linking with a RAND-generated ID. Access to this linked file will be restricted only to authorized study staff. Data transfer from Kenya to RAND will be done with only encrypted, password-protected files using Kiteworks.

### Empirical analysis

The primary method to estimate the impacts of Msingi Bora on parental and child outcomes will be based on an Intention-to-Treat analysis (ITT), which corrects for potential biases due to selective participation into treatment and that represents the parameter of interest for cost-benefit analysis [44]. The identification of the ITT parameter is secured with the random assignment of villages to each of the study arms in our experimental design, and the impacts are obtained performing a series of pairwise comparisons of outcomes across treatment arms, depending on the particular hypothesis being tested. However, in the hypothetical case of sample imbalances at baseline due to the small number of villages per treatment arm, we will further check for the robustness of our ITT estimates using a regression approach including key covariates such as children’s age and gender, household socio-demographics, village characteristics and the outcome of interest measured at baseline.

In a scenario of incomplete compliance with the intervention, the ITT parameter represents a lower bound of the estimated impacts of the program. From a policy perspective, it is perhaps more interesting to estimate treatment effects among program participants. In our intervention, we expect about 75% attendance at the sessions, and we anticipate a very low risk of contamination across villages, as sampled villages will be a minimum distance (> 1.5 km) from each other. The average impact among participants, correcting for potential contamination, is captured by the Local Average Treatment Effect parameter (LATE), obtained using a Two-Stage Least Squares regression approach (2SLS). In a First Stage regression, we will use administrative data on participation to evaluate the predictive effect of random assignment to treatment in participation rates across the different treatment arms. In the Second Stage, final outcomes are regressed on predicted participation rates per treatment arm from the first stage to obtain the LATE parameter. As in the case of the ITT estimator, regressions will include covariates such as children’s characteristics and household’s socio-demographics, village characteristics as well as the outcome of interest at baseline.

In order to explore different pathways of change underlying estimated impacts across treatment arms we will use standard Mediation Analysis methods based on Monte Carlo simulations to construct confidence intervals for indirect effects. In its simplest form, indirect effects are understood as the effect that variable X (the intervention) has on outcome Y that is transmitted through a potential mediating variable M [45, 46]. In our analysis, we will test whether and how each potential hypothesized mediator of change captured in our surveys (knowledge, self-efficacy, social support, mental health, family conflict, etc.) mediates the effect of each intervention on parental behaviors. The analysis of indirect effects for child outcomes will also include parental behaviors as part of the potential set of mediators. Thus, parental behaviors will be both a final outcome in these mediation analyses, as well as an intermediate outcome when child outcomes are the final endpoint considered.

### Missing data and attrition

In all our analyses, we will handle missing data and attrition across survey waves by fitting logistic regression models to assess whether a missing observation is random. We will further construct “nonresponse” weights to correct for non-random dropout in all our regressions and in the calculation of standard errors and tests of significance. In order to test for the importance of outliers, we will check for the robustness of our estimates with the full sample by comparing the estimates from this sample with those from a sample cutting the bottom 2% and the top 2% and testing for the significance of this difference.

### Statistical models

We estimate ITT parameters using a multivariate linear regression approach. Let *Y* denote an outcome of interest at the endline or follow-up survey (child outcomes, parental behaviors or mediators); *D* is a dummy variable for the random allocation to one of the treatment arms: Group meetings only (*d*_1_), Mixed model (*d*_2_), Mothers only (*d*_3_), Mothers and Fathers (*d*_4_); *C* is the dummy variable for the random allocation to the control group; and let *X* be a vector of covariates that include children’s age and gender, household socio-demographics, as well as the outcome of interest measured at baseline. The ITT estimate for the impact of being assigned to treatment arm *k* = {1, 2, 3, 4} with respect to the control group is:

1 $$ {\alpha}_k^{ITT}=E\left[Y|D={d}_k,\mathrm{X}\right]-E\left[Y|C,\mathrm{X}\right] $$

The ITT impact of being assigned to treatment arm *k* vs. *k*^′^ is:

2 $$ {\alpha}_{k,k\prime}^{ITT}=E\left[Y|D={d}_k,\mathrm{X}\right]-E\left[Y|D={d}_{k\prime },\mathrm{X}\right] $$

We will test for the robustness of our estimates by putting these elements together in a standard regression approach and can include village characteristics as well as clustered standard errors at the village level, which is the unit of randomization.

Similarly, the LATE parameter estimating the average treatment on participants of treatment arm *k* with respect to the control group is captured by a Two-Stage Least Squares model through the following specification:

3 $$ {\alpha}_k^{LATE}=\frac{E\left[Y|D={d}_k,\mathrm{X}\right]-E\left[Y|C,\mathrm{X}\right]}{E\left[{P}_k|D={\mathrm{d}}_k,\mathrm{X}\right]-E\left[{P}_k|C,\mathrm{X}\right]} $$

In equation (3), *P*_*k*_ is a variable representing how many sessions an individual actually attended in treatment arm *k* as measured through our administrative attendance records; *E*[*P*_*k*_| *D* = *d*_*k*_, X] is the expected number of sessions that households randomly assigned to treatment arm *k* actually would attend and therefore represents a measure of compliance with treatment among those invited to participate; and *E*[*P*_*k*_| *C*, X] denotes the expected number of sessions that households in the control group receive treatment *k*, which we predict to be zero. Note that this specification also assumes that households assigned to another treatment group *k*^′^ cannot attend the sessions of treatment group *k*. That is, $E\left({P}_k|D={d}_{k^{\prime }},\mathrm{X}\right)=0$. The LATE parameter recovering the impact of participating in treatment arm *k* rather than *k*^′^ is

4 $$ {\alpha}_{k,k\prime}^{LATE}=\frac{E\left[Y|D={d}_k,\mathrm{X}\right]-E\left[Y|D={d}_{k\prime },\mathrm{X}\right]}{E\left[{P}_k|D={d}_k,\mathrm{X}\right]} $$

In a simplified set-up with *P*_*k*_ as a dummy variable indicating any compliance or not, we can estimate richer versions of [3] and [4] that would recover the LATE parameter for any attendance. Similar to the ITT case, in order to recover these parameters from 2SLS regressions we will control for village characteristics and will cluster standard errors at the village level.

### Mediation analysis

Finally, we will conduct our analysis of potential mediators of behavioral change and child outcomes following the Monte Carlo simulation method presented by Preacher and Selig [45]. In a standard mediation model where the outcome of interest is *Y* and the mediating factor is *M*, the goal is to estimate the magnitude and significance of the intervention’s indirect effect (*a* ∗ *b*) as opposed to the direct effect (*c*) from the following model:

5 $$ Y={b}_0+\mathrm{b}M+ cD+\varepsilon $$

6 $$ M={a}_0+ aD+u $$

While our modeling approach presented here relates mediators and treatment to outcome variables in a basic multivariate linear regression approach, we will also explore alternative specifications adding non-linearities such as interactions between treatment and mediator variables.

Following our theory of change, we can investigate the pathways through which one of our intervention arms influences changes in a specified outcome of interest. For example, we can explore if parental behaviors such as stimulation or nutrition practices (*Y*) are affected through changes in mediators of change (*M*) such as knowledge, self-efficacy, social support, mental health, or intra-household conflict. To do this, we will perform the following steps. First, we will run regressions such as Equation (6) for each mediator of change on the dummy variable for the treatment group of interest, controlling for the vector of child and household characteristics to estimate the coefficient $\widehat{a}$ for each mediator. Second, we will run regressions for each type of behavior on the dummy variable for treatment status and on one particular mediator variable of interest, controlling for the same control variables as in the first step, to estimate the coefficient $\widehat{b}$. Using the unstandardized regression coefficient from step one (the a path) and the unstandardized regression coefficient of the respective mediating variable from step two (b path) as well as their squared standard errors, we will compute the 95% Monte Carlo confidence intervals for the indirect effect $\left(\widehat{a}\ast \widehat{b}\right)$ based on a very large number of repetitions. An interval that does not include zero indicates a significant indirect effect of that particular mediating variable for that particular behavior. In order to assess the total indirect effect including all the relevant mediators for one particular behavior of interest, we look at the Monte Carlo confidence intervals using the a paths and b paths from all mediators that resulted to be significant individually but now included together in the same regression model, as in Equation (6).

### Multiple outcomes and multiple hypothesis testing

Our set of outcomes is very large and thus we have multiple hypothesis tests with potentially correlated measures of child outcomes, parental behaviors and mediators of change. To test for the robustness of our estimated impacts for individual outcomes from independent hypothesis tests, we will adopt two approaches. First, we will test for the significance of three different families of outcomes: i) child health and developmental measures; ii) parental stimulation and health behaviors; iii) mediators of behavioral change. We will then construct mean standardized treatment effect estimates to permit us to make summary statements about the overall effects of each intervention arm on each family of outcomes. The mean standardized treatment effect estimates the average of the normalized treatment effects obtained from a seemingly unrelated regression (SUR) where each dependent variable is one of the individual measures belonging to one particular family, for example, child cognitive development as a part of the family “child outcomes” [47].

Second, since we are interested in the testing for robustness of individual estimates to potentially correlated outcomes, we will use will use the Romano Wolf approach, a stepwise multiple testing procedure that asymptotically controls the family-wise error rate [48]. In this method, each coefficient is tested in a step-down fashion using bootstrapping methods allowing for the t-statistics of outcomes to be mutually dependent. Critical values are constructed based on the maximum t-statistic across all outcomes included in each iteration of the test. In the first iteration, all outcomes are included. In further iterations, only outcomes that did not pass the previous test are included.

### Heterogeneous effects

Given the complexity of our experiment and the number of hypothesized channels through which Msingi Bora may affect a variety of outcomes, it is difficult to ex ante hypothesize all possible heterogeneous effects. However, in order to inform the design of targeted policies and address equity-efficiency considerations by remediating socio-economic gaps in child development, it is important to understand whether our interventions are more effective in more disadvantaged households, as well as among younger children. Therefore, we plan to test for the following dimensions of heterogeneous treatment effects: i) by age of the child; ii) by parental age, in particular, differencing between adolescent and non-adolescent mothers; iii) by parental education and assets; iv) by child development outcomes measured at baseline; and v) by child gender.

### Adverse events

Interviews, surveys, and the ECD program are low-risk, and therefore adverse events (AEs) are very unlikely and any experienced AEs will be likely due to factors unrelated to the study. However, there may be adverse consequences to participation that were unintended or unexpected. In these instances, we rely on local monitoring by study team members for reports of adverse events. Study staff will intervene as necessary, assess the participant’s state, and develop an appropriate plan. Incident reports will be written within one business day and study investigators will inform the IRBs of all AEs.

### Dissemination of results

Study findings will be disseminated to researchers via peer-reviewed publications and sharing of findings at conference presentations. In the final year of the project, research team members plan local dissemination workshops to share findings within Kenya with the County Health Management Teams, County Executive Member of Education and Local Administrative Leaders.

## Discussion

Our study’s objective is test for the most effective and cost-effective model of delivery for an integrated ECD intervention to improve child developmental outcomes among young children in rural Kenya. Several challenges and limitations might prevent us from achieving all of the goals of our study.

One potential challenge of our study is that we may not detect impacts at the two-year follow up survey on either originally targeted children or their younger siblings via spillover effects. There are no current examples of a group-based ECD intervention that has demonstrated sustained impacts, and we recognize this is a risk of our study. However, our rich survey measures can potentially shed light on the mediating pathways of change at each time point in our study (baseline, endline, and follow-up), which may help to explain any fade out of impacts if observed.

We will also face several challenges regarding measurement. For example, the application of the Bayley III scale to assess child development is logistically difficult and expensive because it involves direct assessment with children using a complete kit of manipulatives. This requires the child to be in the appropriate mood for the test and the presence of the mother to comfort or breast feed the child. We will implement an extensive training program of the survey interview team and pretest the measures and logistics in an outside sample of mothers and children to establish test-retest and inter-rater reliability measures prior to full field implementation.

Finally, although the pilot study did not identify a significant risk of participant contamination, this remains a possible risk in a cRCT of this nature. CHVs catchment areas will be mapped and selected villages will not overlap to secure that households in control villages do not attend the sessions in contiguous treatment villages, as well as households assigned to a village belonging to one treatment arm do not attend another the sessions of another village belonging to another treatment arm. However, this risk cannot be eliminated entirely. Thus, our sampling strategy will incorporate mapping villages separated by a healthy distance, with the expectation that the high costs of travelling to more distant treatment villages will outweigh the perceived benefit of participating in the intervention. Moreover, we will work closely with SWAP during the sample stage to identify concurrent interventions from other NGOs in the pool of pre-selected villages to avoid overlapping whenever possible, and when it is not possible, to document those interventions to incorporate this information to our statistical analyses.

## Abbreviations

2SLS: Two Stage Least Squares
BL: Baseline
CESD: Center for Epidemiologic Studies Depression Scale
CHV: Community Health Volunteer
cRCT: Cluster randomized controlled trial
DSI: Daily Stress Index
ECD: Early childhood development
EL: Endline
FCI: Family Care Indicators
FU: Follow up
HOME: Home Observation for Measurement of the Environment
HSPC: Human Subjects Protection Committee
ITT: Intention to treat
LATE: Local average treatment effect
LMIC: Lower Middle Income Country
LSNS: Lubben Social Network Scale
MB: Msingi Bora
MDAT: Malawi Development Assessment Tool
MUERC: Maseno University Ethical Review Committee
NGO: Non-governmental organization
NICHD: National Institute for Child Health and Human Development
SEPTI-TS: Self-Efficacy for Parenting Tasks Infant-Toddler Scale
SSA: Sub-Saharan Africa
SUR: Seemingly Unrelated Regression
SWAP: Safe Water and AIDS Project
